# Polymorphisms in *CaSR* and *CLDN14* Genes Associated with Increased Risk of Kidney Stone Disease in Patients from the Eastern Part of India

**DOI:** 10.1371/journal.pone.0130790

**Published:** 2015-06-24

**Authors:** Manalee Guha, Biswabandhu Bankura, Sudakshina Ghosh, Arup Kumar Pattanayak, Saurabh Ghosh, Dilip Kumar Pal, Anurag Puri, Anup Kumar Kundu, Madhusudan Das

**Affiliations:** 1 Department of Zoology, University of Calcutta, Kolkata, West Bengal, India; 2 Human Genetics Unit, Indian Statistical Institute, Kolkata, West Bengal, India; 3 Department of Urology, Institute of Post Graduate Medical Education & Research, Kolkata, West Bengal, India; University of Calcutta, INDIA

## Abstract

Kidney stone disease (KSD) is a major clinical problem imposing a large burden for both healthcare and economy globally. In India, the prevalence of kidney stone disease is rapidly increasing. This study aimed to evaluate the association between genetic defects in vitamin D receptor *(VDR)*, calcium sensing receptor *(CaSR)* and claudin 14 *(CLDN14)* genes and kidney stone disease in patients from eastern India. We enrolled 200 consecutive kidney stone patients (age 18–60 years) (cases) and their corresponding sex and age matched 200 normal individuals (controls). To identify genetic variants responsible for KSD, we performed sequence analysis of *VDR*, *CaSR* and *CLDN14* genes. Four non-synonymous (rs1801725, rs1042636, rs1801726 and rs2228570), one synonymous (rs219780) and three intronic single nucleotide polymorphisms (SNPs) (rs731236, rs219777 and rs219778) were identified. Genotype and allele frequency analysis of these SNPs revealed that, rs1801725 (Ala986Ser), rs1042636 (Arg990Gly) of *CaSR* gene and rs219778, rs219780 (Thr229Thr) of *CLDN14* gene were significantly associated with KSD. Serum calcium levels were significantly higher in subjects carrying 986Ser allele and calcium excretion was higher in subjects bearing 990Gly allele. In conclusion, rs1801725, rs1042636, rs219778 and rs219780 SNPs were associated with kidney stone risk in patients from the eastern part of India.

## Introduction

Kidney stone is a solid crystal aggregation formed in the kidneys from dietary minerals in the urine. Kidney stones are often a painful experience associated with hematuria and if severe or chronic, damage to kidney tissue and renal failure occur [1]. In India, kidney disease is a rising incidence and major problem for healthcare and the economy. Kidney stone formation is a multi-factorial urologic disorder resulting from the combined influence of epidemiological, biochemical and genetic risk factors [2].

The tendency of stone formation is largely attributed to primary renal calcium leak, excessive calcium absorption or an imbalance between bone resorption and formation. Vitamin D receptor *(VDR)* plays an important role in regulating calcium homeostasis by affecting bone resorption and increasing calcium absorption [3]. It has been reported that the intestine, bones and kidneys of genetic hypercalciuric stone-forming rats exhibited increased numbers of vitamin D receptor [4]. A growing amount of epidemiological evidence has suggested that allelic variation of *VDR* gene may be involved in the etiology of kidney stone disease [5]. Several polymorphisms have been identified in the *VDR* gene. Among them four (Apa I, Bsm I, Taq I and Fok I) are particularly studied throughout the globe but with controversial results.

Parathyroid hormone is the main regulator of minute to minute calcium balance and a key regulator of its release iscalcium sensing receptor *(CaSR)*, a G-protein coupled receptor expressed in the parathyroid gland and renal tubular cells [6]. Its activation induced increased calcium excretion in the kidney [7]. In accordance with this, mutation in the *CaSR* gene has been shown to cause abnormalities in blood calcium ion (Ca^2+)^ levels. The presence of an activating and inactivating mutation of *CaSR* gene cause autosomal dominant hypocalcemia or familial hypocalciuric hypercalcemia respectively [8]. Three single-nucleotide polymorphisms causing nonconservative amino acid changes have been described on exon 7, encoding the intracellular domain of CaSR. The most common SNPs, substitution of alanine to serine at codon 986 is associated with increased serum calcium concentration and reduced calcium excretion [9]. Another two frequent SNPs (Arg990Gly and Gln1011Glu) clustered with Ala986Ser. Subsequent association studies between different *CaSR* polymorphisms and urinary calcium have been controversial, resulting in positive [8,10,11] or negative findings [12,13].

A recent genome-wide association study (GWAS) mapped the entire human genome and found SNPs in claudin 14 *(CLDN14)* that was strongly associated with kidney stone [14]. In this study rs219780(C) and rs219781(C) had increased risk of developing kidney stones. CLDN14 is a tight junction membrane protein that is expressed in the epithelia and endothelia and form paracellular barriers and pores that determine tight junction permeability [15]. CLDN14 decreases the permeability of Ca^2+^ through tight junctions. Earlier study has reported that CLDN14 expression is strongly upregulated by activation of CaSR and dysregulation of renal CaSR-CLDN14 pathway could contribute significantly to the development of kidney stone [16].

Recent evidence suggests that the genetic basis of several diseases in Indians might be different from that of Europeans [17,18], this scenario may be due to differences in the risk allele frequency and linkage disequilibrium pattern. Hence, candidate genes of a disease in other populations need to be evaluated for their role in the Indian population. There are few studies that reported the association between *VDR* and kidney stone patients from India. Till date, there is no prior report that elucidates the potential role of *CaSR* and *CLDN14* gene. In this study, we investigated the pathogenetic role of *CaSR*, *CLDN14* and *VDR* genes in kidney stone patients from the Indian population.

## Materials and Methods

### Study Participants

200 patients with kidney stones were recruited from Seth Sukhlal Karnani Memorial hospital (SSKM) & Institute of Post Graduate Medical Education & Research, Kolkata, India in the period of May, 2013-March, 2014. A patient was defined stone former when a stone was observed at ultrasound or X-ray of the kidney or he reported a spontaneous urinary stone emission or a history of surgical stone removal. Patients, who had to have at least one calcium kidney stone, were included. A kidney stone was defined as a calcium stone when chemical or infrared spectrometric analysis showed it was made of calcium oxalate or calcium phosphate or mixed of calcium oxalate and calcium phosphate or mixed of calcium oxalate and calcium phosphate in addition with some other groups like ammonium, magnesium. Patients taking any drug affecting electrolyte or citrate handling (steroids, vitamin D, etc.) were excluded. Other exclusion criteria were endocrine or other disorders in addition to stone disease, abnormal serum creatinine, abnormal serum electrolyte concentration, recurrent urinary infections, evidence of cystic disorders of the kidney and nephropathy etc.

200 age and sex matched healthy subjects were recruited as controls from the same community. They had a negative personal and familial history of kidney stone, normal serum creatinine and calcium concentrations and no evidence of diseases at physical examination. All participants in this study had given written consent. The study protocol was approved by the ethical committee of Institute of Post Graduate Medical Education & Research, Kolkata, India.

### Biochemical parameters

In both stone formers and healthy controls, we measured the serum concentrations of creatinine, serum concentrations of calcium and 24-hour urine excretions of calcium, oxalate, citrate, potassium, phosphate and urate. The urinary pH was measured in stone formers and healthy controls in fresh morning urine samples collected after overnight fasting.

### Analytic procedures used in the serum and urine measurements

Urine was collected in sterile container using 5ml of concentrated HCl as preservative. Whole sample was initially shacked well and was diluted in distilled water (1:20) and finally creatinine was estimated by using modified Jaffe’s reaction and the instrument used was XL-600 analyser (Erba Mannheim, U.S.A.). The quality of the assay was evaluated by internal quality control system. Serum calcium and urinary total calcium were estimated by using arsenazo iii method and instrument used was XL-600 analyser (Erba Mannheim, U.S.A.). Urinary oxalate levels were analyzed by ion chromatography. Urinary Citrate estimation was done using the colorimetric method based on oxidation of citric acid in urine to pentabromacetone and the absorbance read at 445 nm [19]. Estimation of urinary potassium was done by ion selective electrode electrolyte analyser (ISE) by using Easylyte Plus (Medica Corporation, U.S.A.) instrument. Urate was estimated using uricase, peroxidase method by using XL-600 analyser (Erba Mannheim, U.S.A.). Urinary phosphate was estimated by ammonium molybdenate method using XL-600 analyser (Erba Mannheim, U.S.A.).

### Genotyping

Peripheral blood samples were collected from the patients and normal individuals. Genomic DNA was isolated from leukocytes using the QIAamp Blood Kit (QIAGEN, Hilden, Germany). To identify sequence variants, the entire coding region, exon-intron boundaries and some intronic variants of *VDR*, *CaSR* and *CLDN14* genes were amplified by polymerase chain reaction (PCR) and sequenced. All the primers for the coding and non coding regions of the *VDR*, *CaSR* and *CLDN14* genes were listed in S1 Table. The primers for each gene were designed using integrated DNA technologies and primer3 softwares. PCR amplification was undertaken in a 25μl volume containing 100 ng of DNA, 0.5 μl of each primer (10 mmol/L), 0.5 μl of deoxyribonucleotide triphosphate mix (10 mmol/L; Invitrogen Carlsbad, CA, USA), 1 μl magnesium chloride (50 mmol/L), 2.5 μl of 10x buffer and 0.4 μl of Taq Polymerase (5 units/lμl; Invitrogen). The PCR conditions were as follows: denaturation at 94°C for 3 min followed by 44 cycles of denaturation for 30 s, annealing at 58°C-60°C for 45 s, extension at 72°C for 45 s, and final extension at 72°C for 5 min. Forward and reverse strand sequencing was carried out using the big dye terminator kit (Applied Biosystems, Foster City, CA, USA) on an automated DNA capillary sequencer (Model 3700; Applied Biosystems). Sequence alignment between sequences of case and control individuals was performed to find the best matching piecewise (local) or global alignments of two query sequences using ClustalW program.

### Statistical methods

Allelic and genotypic associations of each SNP were tested by using chi-squared or Fisher exact test where appropriate. Hardy-Weinberg equilibrium of each SNP in the case and control individuals were also examined using a χ2 test. To calculate any statistically significant difference of continuous independent variables like age, serum calcium within the control and patient groups, we used Student *t*-test. Mann-Whitney *U* test was used to analyze non-parametric variables. All tests were done using Graphpad Instat software (Graphpad Instat software, San Diego, CA) and SNPassoc version 1.8‐1 software (Catalan Institute of Oncoloy, Barcelona, Spain). The odds ratio and 95% confidential intervals were also calculated using the same software. Linkage disequilibrium (LD) pattern of SNPs in three genes was analyzed using Haploview 4.2. A Bonferroni correction was applied to multiple testing. Power was estimated using genetic power calculator [20].

The combined effect of the four SNPs on the risk of KSD was determined through allele dosage analysis by categorizing the subjects based on the number of “effective” risk alleles. We computed the “effective” number of risk alleles harbored by each individual using a weighted score:

k∑i=1kWiRi∑i=1kWi

Where, k is the number of SNPs, R_i_ is the number of risk alleles at the i^th^ locus and W_i_ is the logarithm of the odds Ratio (OR) of the risk allele at the i^th^ locus, i = 1,2,…, k. The analysis included only those individuals in whom genotypes at all four SNPs were available. Since the effect sizes (defined by the SNP-specific ORs) were not uniform, the allele dosage score of an individual was computed by the weighted mean of the proportion of risk alleles at the four SNPs (i.e., two for two risk alleles, one for one risk allele, and 0 for no risk allele) with weights as the relative log ORs of different SNPs. The “effective” number of risk alleles was obtained as the allele dosage score multiplied by 4 (number of SNPs). Considering subjects with two or fewer numbers of “effective” risk alleles as the reference group, ORs and *P* values for every unit increase in the number of “effective” risk alleles were calculated.

## Result

The basal characteristics and clinical data of the subjects are presented in Table 1.The mean ± SD age of patients was 39.93±11.37 years (range 18–60 years) and 66.5% of them were males and 33.5% were females. There was a high frequency of occurrence of kidney stone among males than that of females. The mean ± SD of BMI was 22.11± 1.19 kg/m^2^. We did not find overweight and obesity in our study samples. Family history of kidney stone was present in 47 patients (23.5%). Of the biochemical parameters, serum creatinine and 24 hours urinary calcium excretion showed significant difference between case and control groups (Table 1).

**Table 1:** Clinical characteristics of the study subjects.

Characters	Patient(n = 200)	Control(n = 200)	*p*-value
Age (years)	39.93±11.37	38.13±10.16	0.1249
Gender			
Male	133 (66.5%)	122 (61.0%)	
Female	67 (33.5%)	78 (39.0%)	0.298
BMI (kg/m^2^)	22.11±1.19	22.20±1.34	Ns
Serum creatinine (mg/dl)	1.21±0.477	0.714±0.096	**<0.001**
Serum calcium (mg/dl)	9.42±0.32	9.46±0.238	Ns
Urinary calcium excretion (mmol/24 h)	7.87±0.59	4.04±0.68	**<0.001**
Urinary Oxalate (mg/24 h)	28.11±3.07	27.51±3.11	Ns
Urinary Citrate (mmol/24 h)	2.58±0.96	2.77±1.21	Ns
Urinary potassium (mmol/24 h)	64.21±4.77	64.59±4.61	Ns
Urinary Phosphate (mmol/24 h)	27.45±4.29	26.81±3.33	Ns
Urinary Urate (mmol/24 h)	2.89±0.47	2.78±0.86	Ns
Spot urine pH	5.72±0.31	5.79±0.55	Ns

Values given are averages ± SD.

We investigated polymorphisms in 200 kidney stone patients and 200 controls by direct sequencing of *VDR*, *CaSR* and *CLDN14* genes. Four non-synonymous, one synonymous and three intronic polymorphisms were identified (Table 2A & 2B). The genotype distributions at all the SNPs were in HWE except rs731236 of *VDR* gene.

**Table 2:** Allele distribution of *CaSR*, *VDR* and *CLDN14* gene polymorphisms in the study.

Gene	SNP	Allele	Allele frequency		Odds Ratio (95% CI)	*p*-value
			Case	Control		
*CaSR*	rs1801725:Ala986Ser	G	0.79	0.90	Reference	
		T	0.21	0.10	**2.54(1.69–3.81)**	**<0.001**
	rs1042636:Arg990Gly	A	0.68	0.82	Reference	
		G	0.32	0.18	**2.21(1.58–3.07)**	**<0.001**
	rs1801726:Glu1011Gln	C	0.97	0.975	Reference	
		G	0.03	0.025	1.10(0.46–2.63)	1.00
*VDR*	rs2228570:Met1ThrFok1	C	0.68	0.73	Reference	
		T	0.32	0.28	1.25(0.93–1.70)	0.164
	rs731236: taq1, t>c	T	0.48	0.47	Reference	
		C	0.52	0.54	0.94(0.71–1.24)	0.723
*CLDN14*	rs219777:c>t	C	0.89	0.93	Reference	
		T	0.11	0.07	1.90(1.16–3.11)	0.013
	rs219778:t>c	T	0.85	0.94	Reference	
		C	0.16	0.06	**3.01(1.82–4.96)**	**<0.001**
	rs219780:Thr229Thr	G	0.84	0.92	Reference	
		A	0.16	0.08	**2.19(1.40–3.43)**	**0.001**

chi square test was used to compare the allele frequencies between cases and controls.

### *VDR* gene polymorphisms

Genetic analysis of *VDR* gene in both case and control groups, showed two single nucleotide changes, which include one non-synonymous change (rs2228570; p.Met1Thr) and one intronic change (rs731236). The genotype frequencies and allelic distribution of these polymorphisms are given in Table 2. The p.Met1Thr change is a functional polymorphism identified in the start codon of the protein. A T/C polymorphism (rs731236) was also detected in ninth intron of this gene. Dominant model provided a slightly significant association (*p-*0.077) for rs2228570 (p.Met1Thr) and rs731236 (*p-*0.060). However, the significant association of rs2228570 and rs731236 was lost after the Bonferroni correction for multiple comparisons.

### *CaSR* gene polymorphisms

Three different amino acid changes (rs1801725:Ala986Ser, rs1042636:Arg990Gly, rs1801726:Glu1011Gln) in the *CaSR* gene were identified through the sequencing of all coding exons of the gene from 200 patients and 200 controls. The allelic distribution and genotype frequencies of these polymorphisms are given in Table 2 and Table 3 respectively.

**Table 3:** Genotype distribution of *CaSR*, *VDR* and *CLDN14* gene polymorphisms in the study.

Gene	SNP	Genotype	Case(n = 200)	Control(n = 200)	Odds ratio (95% CI)	*Adjusted Odds ratio (95% CI)	*p*-value
*CaSR*	rs1801725: Ala986Ser	GG	116	162	Reference	Reference	
		GT	82	37	GG vs GT: 3.10(1.96–4.88)	**GG vs GT: 3.07(1.94–4.86)**	**<0.001**
		TT	2	1	GG vs TT: 2.79(0.25–31.17)	GG vs TT: 2.63(0.23–21.70)	0.745
					GG vs GT+TT:3.09(1.97–4.85)	**GG vs GT+TT:3.06(1.94–4.82)**	**<0.001**
	rs1042636:Arg990Gly	AA	86	130	Reference	Reference	
		AG	99	69	AA vs AG: 2.17(1.44–3.27)	**AA vs AG: 2.18(1.44–3.31)**	**<0.001**
		GG	15	1	AA vs GG: 22.67(2.94–174.82)	**AA vs GG: 20.76(2.86–160.73)**	**<0.001**
					AA+AGvsGG:16.14(2.11–123.37)	**AA+AGvsGG:14.87(1.94–114.26)**	**<0.001**
	rs1801726:Glu1011Gln	CC	189	190	Reference	Reference	
		CG	11	10	CC vs CG: 1.11(0.46–2.67)	CC vs CG: 1.17(0.84–2.84)	0.73
		GG	0	0			
*VDR*	rs2228570:Met1Thr:Fok1	CC	78	98	Reference	Reference	
		CT	115	90	CC vs CT: 1.61(1.07–2.41)	CC vs CT: 1.53(1.02–2.31)	0.060
		TT	7	12	CC vs TT: 0.73(0.28–1.95)	CC vs TT: 0.71(0.26–1.90)	0.701
					CC vs CT+TT:1.50(1.01–2.24)	CC vs CT+TT:1.43(0.96–2.14)	0.077
	rs731236:taq1, t>c	CC	60	77	Reference	Reference	
		CT	82	58	CC vs CT:1.66(0.96–2.88)	CC vs CT:1.90(1.17–3.08)	0.020
		TT	58	65	CC vs TT:3.28(0.34–31.89)	CC vs TT:1.13(0.69–1.85)	0.656
					CC vs CT+TT:1.73(1.01–2.95)	CC vs CT+TT:1.48(0.97–2.25)	0.060
*CLDN14*	rs219777:c>t	CC	159	174	Reference	Reference	
		CT	38	25	CC vs CT:1.66(0.96–2.88)	CC vs CT:1.66(0.96–2.89)	0.110
		TT	3	1	CC vs TT:3.28(0.34–31.89)	CC vs TT:3.33(0.34–32.63)	0.550
					CC vs CT+TT:1.73(1.01–2.95)	CC vs CT+TT:1.73(1.01–2.69)	0.040
	rs219778:t>c	TT	141	178	Reference	Reference	
		TC	56	21	TT vs TC:3.37(1.95–5.82)	**TT vs TC:3.46(1.99–6.00)**	**<0.001**
		CC	3	1	TT vs CC:3.79(0.39–36.80)	TT vs CC:3.48(0.36–33.96)	0.452
					TT vs TC+CC:3.39(1.98–5.79)	**TT vs TC+CC:3.46(2.01–5.94)**	**<0.001**
	rs219780:Thr229Thr	GG	139	169	Reference	Reference	
		GA	58	30	GG vs GA:2.35(1.43–3.85)	**GG vs GA:2.58(1.56–4.28)**	**<0.001**
		AA	3	1	GG vs AA:3.65(0.38–35.46)	GG vs AA:4.00(0.41–39.10)	0.350
					GG vs GA+AA:2.39(1.47–3.89)	**GG vs GA+AA:2.63(1.60–4.32)**	**<0.001**

*Odds ratio were adjusted for age, sex, and BMI.

chi square test was used to compare the genotype frequencies between cases and controls.

**Fig 1:**
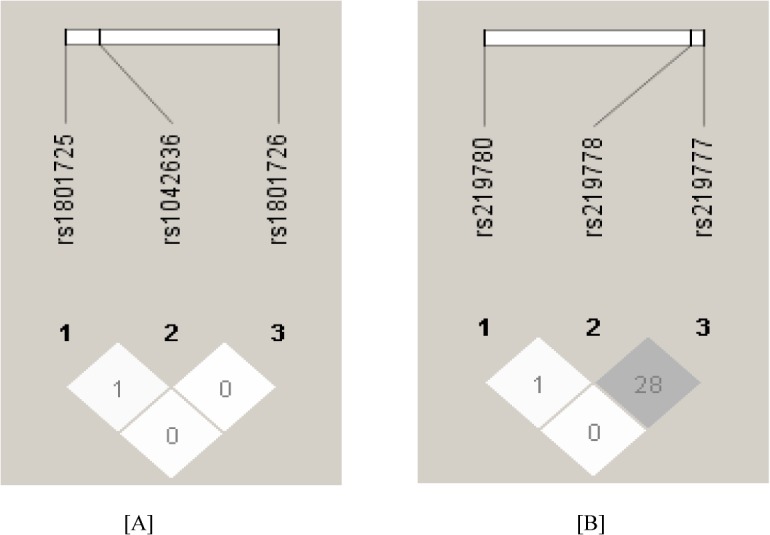
[A] & 1[B] Linkage disequilibrium (LD) pattern (r2) of the three SNPs in CaSR gene and three SNPs in CLDN14 gene respectively in case and control groups. The LD between the SNPs is measured as r2 and shown in the diamond at the intersection of the diagonals from each SNP. r2 = 0 is shown as white, 0 < r2 <1 is shown in gray and r2 = 1 is shown in black.

No linkage disequilibrium was observed among the 3 single nucleotide polymorphisms (Fig 1A). Among three polymorphism two (rs1801725:Ala986Ser, rs1042636:Arg990Gly) are strongly associated with kidney stone disease. Therefore, our results suggest that for rs1801725 (Ala986Ser) T allele is the risk allele (*p*<0.001; Odds ratio = 2.54; 95% CI: 1.69–3.81) towards the development of kidney stone disease (Table 2). Simultaneously, when we combined the variant TT genotype with the GT genotype (i.e., GT+TT), assuming a dominant genetic model, we observed 3 fold increased risk with the combined genotype GT+TT compared with the GG genotype (*p*<0.001; Odds ratio = 3.06; 95% CI: 1.94–4.82).

The Arg990Gly (rs1042636) amino acid change, a rare gain of function variant of *CaSR* gene is strongly associated with kidney stone disease in our study population. The genotype frequency of Arg990Gly (rs1042636) polymorphism was significantly different between case and control groups (p < 0.001). Compared with the wild type genotype AA, the variant genotype GG was associated with 20 fold increased risk (*p*<0.001; Odds ratio = 20.76; 95% CI: 2.86–160.73) for kidney stone patients. Similarly, the variant allele G frequency was significantly higher in kidney stone patients (32%) and conferred susceptibility to kidney stone (*p*<0.001; Odds ratio = 2.21; 95% CI: 1.58–3.07). Our result strongly suggested that individual carrying G allele or GG genotype is more predominant to develop kidney stones in our population.

In addition, we identified another polymorphism, rs1801726 (Glu1011Gln) in exon7 of *CaSR*gene. Homozygote genotype for this variant was not found in our study subjects and there was no association.

### Association between polymorphism (rs1801725 and rs1042636) of *CaSR* gene and phenotypes in stone formers and healthy controls

We evaluated the association between these SNPs and the quantitative variables of KSD in our study population. Our result revealed that, serum calcium levels were significantly higher in individuals carrying 986Ser allele (Table 4). Further, when the interaction between rs1042636 and quantitative variables were assessed, urinary calcium excretion remained significantly higher in patients carrying 990Gly allele (Table 4).

**Table 4:** Values of quantitative traits were compared in stone formers and healthy controls with rs1801725 and rs1042636 SNPs of *CaSR* gene.

Characters	Case						Control					
	rs1801725 (Ala986Ser)			rs1042636 (Arg990Gly)			rs1801725 (Ala986Ser)			rs1042636 (Arg990Gly)		
	GG (n = 116)	GT+TT (n = 84)	*p* value	AA(n = 86)	AG+GG (n = 114)	*p* value	GG(n = 162)	GT+TT (n = 38)	*p* value	AA(n = 130)	AG+GG (n = 70)	*p* value
Age	38.84±11.12	41.43±11.60	0.13	39.14±11.57	40.76±11.17	0.334	37.99±10.38	38.71±9.09	0.677	37.35±10.32	39.57±9.68	0.064
Serum creatinine (mg/dl)	1.14±0.27	1.31±0.65	0.11	1.19±0.32	1.23±0.56	0.843						
Serum calcium (mg/dl)	9.20±0.20	9.72±0.18	**<0.001**	9.43±0.32	9.41±0.32	0.869	9.45±0.21	9.64±0.25	**<0.001**	9.47±0.23	9.51±0.23	0.179
Urinary calcium Excretion (mmol/24h)	7.97±0.63	7.83±0.50	0.09	7.58±0.57	8.10±0.51	**<0.001**	4.09±0.68	3.80±0.63	0.125	4.04±0.71	4.02±0.62	0.882
Urinary oxalate (mg/24h)	28.47±3.37	27.63±2.54	0.180	28.04±3.14	28.17±3.03	0.685						
Urinary citrate (mmol/24 h)	2.44±0.62	2.41±0.75	0.604	2.42±0.64	2.43±.70	0.826						
Urinary potassium (mmol/24 h)	64.08±4.78	64.16±4.76	0.956	63.88±4.99	64.30±4.59	0.548						
Urinary phosphate (mmol/24 h)	27.37±4.67	27.57±3.73	0.956	27.87±5.02	27.51±3.63	0.892						
Urinary urate (mmol/24 h)	2.88±0.50	2.89±0.43	0.367	2.94±0.48	2.85±0.47	0.262						

Values given are averages ± SD.

### Combined effect of rs1801725 and rs1042636 polymorphism of *CaSR* gene with KSD risk

To further elucidate the combined effect of both polymorphisms, we considered individuals carrying both risk alleles (T of rs1801725 and G of rs1042636) and compared with individuals carrying single risk allele and no risk allele. We found that individuals carrying both risk alleles showed 2 fold increase risk (*p-* 0.03; Odds ratio = 2.02; 95% CI: 1.07–3.80) for the development of kidney stone disease compared to individuals carrying single risk allele and 5 fold increased risk (*p*<0.001; Odds ratio = 5.09; 95% CI: 2.65–9.77) compared to individuals having no risk allele.

### *CLDN14* gene polymorphisms

To identify variant (s), we sequenced the exons and their immediate flanking regions of *CLDN14* gene in 200 cases and 200 controls. Two non-exonic (rs219777, rs219778) and one exonic (rs219780:Thr229Thr) SNPs were identified. We were not observed linkage disequilibrium among the 3 SNPs (Fig 1B). After the Bonferroni correction for multiple comparisons, we observed a strong association between the rs219778 (t>c) and rs219780 (Thr229Thr) SNPs and KSD (Table 2 & Table 3). In all genotypes combined, the dominant model of these two SNPs showed very significant association with KSD: OR = 3.46 (95% CI = 2.01–5.94) and P = <0.001 for rs219778, and OR = 2.63 (95% CI = 1.60–4.32) and P = <0.001 for rs219780. No association was observed between the risk SNPs and the serum creatinine, urinary calcium excretion and serum calcium (data not shown).

### Combined effect ofrs219778 and rs219780 polymorphism of *CLDN14* gene with KSD risk

The combine risk of these two SNPs were estimated when we compared individuals carrying both risk alleles (C of rs219778 and A of rs219780) with individuals carrying single risk allele and no risk allele. Our result revealed that individuals carrying both risk alleles showed 9.5 fold increase risk (*p*-<0.001; Odds ratio = 9.50; 95% CI: 2.77–32.56) for the development of kidney stone disease compared to individuals having no risk allele, whereas for individuals carrying single risk allele no significant difference was found.

### Combined effect of rs1801725, rs1042636, rs219778 and rs219780 polymorphism with KSD risk

To estimate the risk for KSD in individuals carrying these 4 risk alleles, we compared individuals carrying 4 risk alleles (T of rs1801725, G of rs1042636, C of rs219778 and A of rs219780) with individuals carrying less number of risk alleles or no risk allele. Our study revealed that individuals carrying 4 risk alleles showed approximately 8 fold increase risk (*p-* 0.04; Odds ratio = 8.29; 95% CI: 1.03–66.93) for the development of kidney stone disease compared to individuals carrying less number of risk alleles or no risk allele. To appraise the correlation between quantitative variables of KSD and these 4 SNPs, we compared the levels of serum calcium and urine calcium in individuals carrying these 4 risk alleles and those carrying less number of risk alleles. Urinary calcium excretion and serum calcium levels were significantly higher in individuals carrying 4 risk alleles in comparison with individuals carrying less number of risk alleles (Table 5).

**Table 5:** Comparison of serum calcium concentration and urinary calcium excretion level with individual carrying 4 risk alleles and individuals carrying less numbers of risk alleles.

Characters	Individual carrying 4 risk alleles (n = 9)	Individuals carrying less numbers of risk alleles (n = 282)	*p* value
Serum calcium (mg/dl)	9.73±0.21	9.46±0.28	**0.005**
Urinary calcium Excretion (mmol/24h)	7.60±1.06	6.14±2.01	**0.014**

Values given are averages ± SD.

### Allele dosage analysis

**Fig 2:**
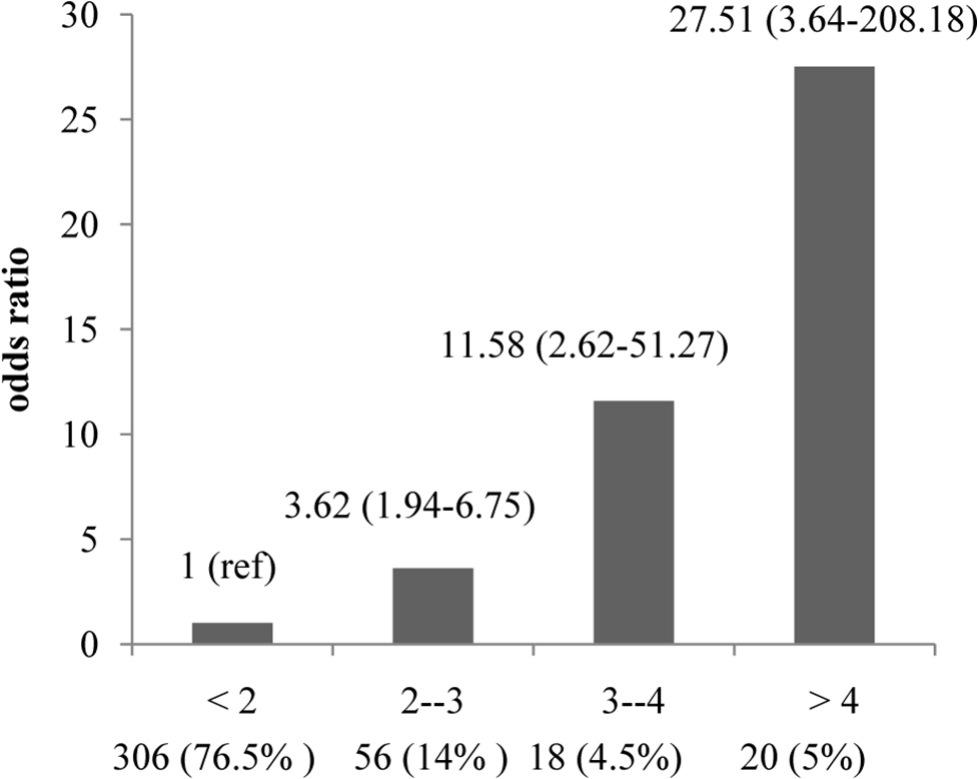
Effect of increase in the number of “effective” risk alleles on the risk of Kidney stone disease in combined samples. OR are plotted on the *y*-axis for the corresponding number of “effective” risk alleles on the *x*-axis. Numbers in parentheses on *x*-axis indicate sample size in each category. <2 group considers as reference group. <2: Individuals having 2 or less “effective” risk alleles. 2- -3: Individuals having 2–3 “effective” risk alleles. 3- -4: Individuals having 3–4 “effective” risk alleles. >4: Individuals having 4 or more “effective” risk alleles.

Allele dosage analysis showed a significantly enhanced risk of KSD with the increase in each unit of “effective” risk allele (Fig 2). Individuals having more than 4“effective” risk alleles (5%) showed 27.5 fold increased risk for KSD in comparison with individuals having less than 2“effective”risk alleles (76.5%).

## Discussion

KSD is a common and complex clinical condition imposing a large economic burden on the healthcare system. Hypercalciuriais the major risk factor for development of a calcium containing stone. An alteration of renal regulations of calcium excretions is the main reason for Hypercalciuria. The G-protein-coupled CaSR which is expressed in the basolateral membrane of thick ascending limb, plays a central role in the regulation of PTH secretion and renal tubular calcium reabsorption in response to blood calcium levels [21]. The CaSR decrease renal tubule calcium reabsorption and cause hypercalciuria by suppressing the activity of calcium sensitive potassium channel. The CaSR gene is also regulated via VDR, and elevation of VDR levels is observed in genetic hypercalciuric stone-forming rats have been implicated in the increased renal CaSR mRNA induced by 1,25 dihydroxy vitamin D3 [22]. On the other hand CLDN14 play an important role in the regulation of renal Ca^2+^ excretion. CLDN14 is also regulated by CaSR signaling. CLDN14 expression would be increased by the activating mutation of CaSR gene and causing nephrocalcinosis [16]. Therefore, these three genes (*VDR*, *CaSR* and *CLDN14*) are considered as candidate gene for KSD. In the present study, we evaluate the potential association of *VDR*, *CaSR* and *CLDN14* genes in patients with KSD in the eastern part of India.

Epidemiologic studies reported that the prevalence and incidence of kidney stones are increasing, although it is still an ambiguous problem for the biologist with very imprecise understanding of its pathogenesis and mode of early detection due to lack of well-established genetic marker. A lot of genetic study has been performed to explore the relationship between SNPs and KSD. Several genetic markers including polymorphisms of genes coding for the *VDR*, interleukin, fibronectin and urokinase [5] have been investigated. The most important gene related to calcium metabolism, *VDR* gene has earned special concern.

Several studies investigated the association between the *VDR* polymorphism and KSD, but the results were conflicting. Some study detected an association between *VDR* polymorphism and KSD [23–25], whereas some study did not confer a significant risk for KSD [3,26,27]. The contradictory results from different study may be attributed to various factors, such as complexity of disease etiology, genetic heterogeneity of disease, ethnicity, differences in population characteristics, including interaction with environmental factors, selection of control group, sample size, and gene–gene and gene–environment interactions [3].

In the present study, we investigated the association of *VDR* polymorphismsin kidney stone patients and controls in Indian population. We found two SNPs (rs2228570: FokI and rs731236: TaqI) and the allele frequencies of these SNPs are C: 73%, T: 28% of rs2228570 (FokI) and T: 47%, C: 54% of rs731236 (TaqI). The allele frequencies of rs2228570 in the present study were different compared to Caucasian and Asians (C: 59.8%, T: 40.2% and C: 59.6%, T: 40.4% respectively) [according to International Hapmap project]. On the other hand the allele frequencies of rs731236 were in between the frequencies of European (T: 60%, C: 40%) and Asian (T: 93%, C: 7%) populations [according to International Hapmap project]. Our results revealed that the frequencies of FokI and TaqI alleles of *VDR* gene did not differ significantly between the patient and control groups. Although a previous study from northern part of India confirms an association between the FokI and ApaI polymorphism and urolithiasis [28], our results are more in line with a report suggesting the lack of association in a Korean population [29]. The frequencies of FokI and TaqI alleles may vary among different populations. This discrepancy of the result may be due to several factors, such as the diverse Indian population with different socio-cultural traditions; different genetic backgrounds between ethnic groups and the linkage disequilibrium pattern between different *VDR* gene polymorphisms has been shown to diverge among different ethnic groups [29].

*CaSR* gene (chr. 3q13.3–21) encodes for a protein of 1078 amino acids expressed in the plasma membrane as a dimer. In our study three polymorphisms have been identified in the intracellular tail of the receptor: Ala986Ser, Arg990Gly and Glu1011Gln. Ala986Ser was the most frequent polymorphic variant in several populations. In this study, we found that 986Ser (T) allele is significantly associated with KSD. A number of studies [30,31] have observed an association between 986Ser (T) allele and higher levels of serum calcium. In agreement with the findings from the other studies, we also found calcium concentrations were significantly higher in individuals carrying 986Ser (T) allele in our study. This finding suggests the inhibitory activity of CaSR on tubular calcium reabsorption and parathyroid hormone secretion be depressed in subjects carrying 986Ser (T) allele. CaSR is sensitive to serum calcium for its location on basolateral membrane of tubular cells. Here, CaSR modulates calcium reabsorption according to the serum calcium levels [10]. Therefore, the increase of plasma calcium was coupled with elevated calcium excretion in kidney stone patients carrying 986Ser (T) allele. Another important polymorphism of *CaSR* gene is Arg990Gly located in Exon 7 and associated with hypercalciuria in patients with and without kidney stones. Results of *in vitro* analysis indicate that the Arg990Gly polymorphism can give rise to a functional gain for the CaSR [10]. Therefore, 990Gly allele increases CaSR sensitivity, which should result in greater inhibition of calcium re-absorption into the cells of the thick ascending limb of the Henle’s loop and cause higher calcium excretion levels [32]. A high calcium excretion levels are prospectively dangerous for the kidney as it enhances the probability of calcium-phosphate precipitation inside the kidney and stone formed [10]. In our study Arg990Gly polymorphism is highly associated with KSD. Individuals carrying G allele and GG genotype have higher risk to developed KSD. In addition, patients who carry 990Gly allele variant have higher urinary calcium levels and may promote to formation of stone in our study group. Our results also showed that, individuals carrying both 986Ser and 990Gly allele have increased the risk of KSD in comparison to individuals carrying 986Ser or 990Gly.

CLDN14 is a membrane protein that regulates paracellular passage of ions and small solutes at epithelial tight junction [33]. The overexpression of claudin-14 in the thick ascending limb of Henle’s loop of the kidney generates a renal phenotype characteristic with hypomagnesemia and hypercalciuria [34]. Polymorphisms of this gene were identified in only one study done by Thorleifsson et al, 2009 in European KSD patients. They found four polymorphisms (rs219778, rs219779, rs219780 and rs219781) in *CLDN14* gene were associated with KSD. In the present study, we found three SNPs (rs219777, rs219778 and rs219780), among them two (rs219778 and rs219780) were significantly associated with KSD. Simultaneously, when we compared between individuals carrying two risk allele (C of rs219778 and A of rs219780) and individuals having no risk allele, approximately 10 fold increased risk has been found for individuals having two risk allele. For establishment of genotype and phenotype correlation, we decided to test correlation between rs219778: rs219780 and biochemical values. No association was observed between the risk variants and serum createnine, urinary calcium excretion, serum calcium levels.

While all the four loci independently predicted the risk for KSD, the risk is increased significantly if all the alleles were present together. A 27.5-fold risk increased among individuals carrying 4 or more “effective” risk alleles compared with individuals with 2 or less “effective” risk alleles. This information added extra influence of these genetic variants on risk for KSD and their likely role in predictive genetic testing.

Our study has several limitations. This study is under powered; elucidation of these results with much larger sample size may help in better understanding of the role of *VDR*, *CaSR* and *CLDN14* gene variants in KSD. Although computed tomography scan is a gold standard for detecting kidney stones, but in our study we classified healthy controls based on ultrasound imaging.

In conclusion, common variants in *CaSR* and *CLDN14* genes are associated with KSD in the eastern part of India. Nevertheless *VDR* does not seem to be a candidate gene of Kidney stone in our study population. Our studies suggest that *CaSR* and *CLDN14* are candidate genes to explain the individual predisposition to calcium kidney stones. Previously published data suggested that both CaSR and CLDN14 were part of a common pathway [16]. Based on this finding, our result suggests an alteration in the CaSR-CLDN14 signalling pathway and it likely contributes significantly to the development of hypercalciuria and the formation of KSD.

## Supporting Information

S1 Table Primers using for amplification of coding and noncoding part of *CaSR*, *CLDN14* and *VDR* gene. (DOCX)
