# Supplementary material for: Polymorphisms in CaSR and CLDN14 Genes Associated with Increased Risk of Kidney Stone Disease in Patients from the Eastern Part of India
Source: PLoS One. 2015 Jun 24;10(6):e0130790. doi: 10.1371/journal.pone.0130790 (PMC4480968; doi:10.1371/journal.pone.0130790)
Supplement: S1 Table — (DOCX) [file pone.0130790.s001.docx]

**S1 Table. Primers using for amplification of coding and noncoding part of *CaSR, CLDN14* and *VDR* gene**

| **Gene Name** | **Primer name** | **Primer sequence** | **Product length(bp)** |
| --- | --- | --- | --- |
| ***CaSR*** | Exon 1 | F:5’- GCCCACCCAAAGGAGTATG -3’ | 502 |
|  |  | R: 5’- TCCTTGGCGATGGAAAGAAG -3’ |  |
|  | Exon 2 | F:5’-TTCCAAAGACTCAAGGACCACCCA-3’ | 680 |
|  |  | R:5’-ACTGCTGCCAGGTGAACGATGATA-3’ |  |
|  | Exon 3 | F:5’-AGCATGCCATGAAGCCAGAGAGTA -3’ | 485 |
|  |  | R: 5’-AAGCCTGCTTCTTCTGATCCTGCT-3’ |  |
|  | Exon 4 | F:5’-ACTCATTCACCATGTTCTTGGTTCT-3’ | 700 |
|  |  | R:5’- GCTGTTGCTAAACCTGTCGC-3’ |  |
|  | Exon 5 | F:5’-ACCGGCAAGTACTTTGGTGTGAAT -3’ | 540 |
|  |  | R:5’-AGATTGCAAAGGCCAGAGAGTTCA -3’ |  |
|  | Exon 6 | F: 5’-TTCTTGTGCCCAAACTCCTCCC -3’ | 565 |
|  |  | R: 5’-TTTCTGTTCCACCTCCATGCCC-3’ |  |
|  | Exon 7A | F: 5’-CCACCACATGTACACTCACACA-3’ | 670 |
|  |  | R: 5’- GGTTGCGGTAGCTTGACG-3’ |  |
|  | Exon 7B | F:5’-TGTCCTCCTGGTGTTTGAGG-3’ | 698 |
|  |  | R:5’-GCTGCTGCTTCTGCCTCT-3’ |  |
|  | Exon 7C | F:5’-GGTCCAGCAGCCTTGGA-3’ | 637 |
|  |  | R:5’-CCTCAGAGGAAAGGAGTCTGG-3’ |  |
| ***CLDN14*** | Exon 1-2 | F:5’-GGGCTCAGCATTCCCATATC-3’ | 528 |
|  |  | R:5’-CTGCTTATGTCCACCCTTGAG-3’ |  |
|  | Exon 3A | F:5’-CATTTCCTTTCTCTCCCTGCTC-3’ | 616 |
|  |  | R:5’-GCCTGGCCAATCTCAAACT-3’ |  |
|  | Exon 3B | F:5’-CTTCTACAACCCGCTGCT-3’ | 618 |
|  |  | R:5’-GCAAGGTTTATTCCTGGATCAC-3’ |  |
|  | 3UTR | F:5’-GGAATAAACCTTGCGGATGTG-3’ | 571 |
|  |  | R:5’-GAGTAGCTGGGATTACAGGTG -3’ |  |
| ***VDR*** | Exon 1 | F:5’- GGCCTATAGGGTGGTTGATT-3’ | 432 |
|  |  | R: 5’- GCACGAACTTCAGCTTTCTC-3’ |  |
|  | Exon 2 | F:5’- GAGGTAGGATTGTGCAGAGAAA-3’ | 539 |
|  |  | R:5’- TCTACCAAGGAACCCTGAGA-3’ |  |
|  | Exon 3 | F:5’-GTATGAGGGCTCCGAAGG-3’ | 500 |
|  |  | R:5’ GAAGATACCACTCACCAAGACC-3’ |  |
|  | Exon 4 | F:5’-CAGCCTAGAGGTGAGAGTGA-3’ | 505 |
|  |  | R:5’- CTGTGCACCTCTTGAGGATC-3’ |  |
|  | Exon 5 | F:5’- CCCGACTCCCTAAAGCC- 3’ | 644 |
|  |  | F:5’- ATCAGAGATCAGGGCCAAG-3’ |  |
|  | Exon 6 | F:5’- CCAGGGAGTAGGGACAGA-3’ | 555 |
|  |  | R:5’- GTGTAGCATCAGCCTCCC- 3’ |  |
|  | Exon 7 | F:5’- TCTGAGTGTCTCTGGCTCTT-3’ | 641 |
|  |  | R:5’- GATTACAGGCACCAGACACC -3’ |  |
|  | Exon 8 | F:5’- GCTACTTCACTTCTGGCCTTG-3’ | 558 |
|  |  | R:5’- CTCTGCCCGCAGTACCT -3’ |  |
|  | Exon 9 | F:5’- GCCTAGACTCCACCTCCT-3’ | 493 |
|  |  | R:5’- GTGTGTGGACGCTGAGG-3’ |  |
|  | Exon 10 | F:5’- TCACCGGTCAGCAGTCATAG-3’ | 548 |
|  |  | R:5’- AGAATGGGCTGGGTGGATAG-3’ |  |
